# Photoracemization‐Based Viedma Ripening of a BINOL Derivative†

**DOI:** 10.1002/chem.201904382

**Published:** 2019-12-12

**Authors:** Giuseppe Belletti, Carola Tortora, Indradevi D. Mellema, Paul Tinnemans, Hugo Meekes, Floris P. J. T. Rutjes, Svetlana B. Tsogoeva, Elias Vlieg

**Affiliations:** ^1^ Radboud University Institute for Molecules and Materials Heyendaalseweg 135 6525 AJ Nijmegen The Netherlands; ^2^ Chair of Organic Chemistry I Department of Chemistry and Pharmacy Friedrich-Alexander-Universität Erlangen-Nürnberg Nikolaus-Fiebiger-Str. 10 91058 Erlangen Germany

**Keywords:** chirality, crystal growth, deracemization, photoracemization, Viedma ripening

## Abstract

Viedma ripening is a deracemization process that has been used to deracemize a range of chiral molecules. The method has two major requirements: the compound needs to crystallize as a conglomerate and it needs to be racemizable under the crystallization conditions. Although conglomerate formation can be induced in different ways, the number of racemization methods is still rather limited. To extend the scope of Viedma ripening, in the present research we applied UV‐light‐induced racemization in a Viedma ripening process, and report the successful deracemization of a BINOL derivative crystallizing as a conglomerate. Irradiation by UV light activates the target compound in combination with an organic base, required to promote the excited‐state proton transfer (ESPT), leading thereafter to racemization. This offers a new tool towards the development of Viedma ripening processes, by using a cheap and “green” catalytic source like UV light to racemize suitable chiral compounds.

## Graphical abstract

**Turn the light on**! The application of UV light as a “green” and cheap catalytic source to induce racemization in a Viedma ripening process is reported. This approach successfully yielded enantiomerically pure crystals of a BINOL derivative, which crystallizes as a metastable conglomerate solvate.

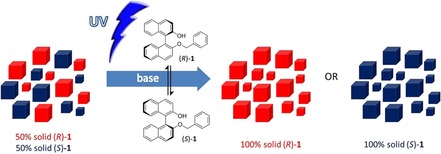

## Introduction

The increasing demand of single enantiomers for industrial applications continues to stimulate the development of feasible and economically viable methodologies to reach enantiopurity.^1^, ^2^ Besides classical resolution techniques and dynamic resolution methods, Viedma ripening has been recognized as a valid deracemization process.^3^, ^4^, ^5^, ^6^, ^7^ Since its discovery in 2005, several conditions and parameters were screened to improve the efficiency of this technique.^1^, ^8^, ^9^, ^10^ Organic compounds, such as amino acids and their derivatives,^11^, ^12^ organometallic complexes^13^ and pharmaceutical intermediates,^4^, ^5^ were successfully deracemized by using Viedma ripening. During the process, a slurry of the conglomerate crystals in a saturated solution is intensively ground to promote breakage and dissolution as well as growth of the crystals. In this way, an enantiopure crystalline final state can be achieved, in up to 100 % yield.^14^, ^15^ The technique is based upon two essential prerequisites: the target molecule has to crystallize as a conglomerate, that is, a mechanical mixture of enantiomerically pure crystals, and suitable racemization conditions have to be applied in solution, to continuously interconvert one enantiomer into the other. Up to now, the Viedma ripening process has had limited applications, given the finite number of molecules which meet the two prerequisites. For finding conglomerates, several strategies have been proposed, but the scope for racemization is still rather limited. Racemization strategies which have been applied to Viedma ripening typically involve base catalysis,^5^, ^15^, ^16^, ^17^ but this limits the operations to acidic chiral carbons bearing protons that can be removed. The development of new racemization methods is therefore important and is the key to extend the applicability of Viedma ripening to several other substrates. Photocatalysis is a powerful tool to promote racemization of photosensitive organic compounds. The application of UV light to molecules having sufficient absorbance in the UV region can lead to racemization by bond breakage or by exciting the molecular orbitals, allowing for rearrangements that are forbidden in the ground state.^18^ Up to now, a few successful examples of resolution through dynamic crystallization of prochiral molecules racemizing under UV light starting from completely achiral conditions have been reported.^19^, ^20^ Here, we aim to extend the application of Viedma ripening to molecules racemizing by photocatalysis, and focus the attention on chiral BINOL derivatives. This class of compounds is widely used as catalysts in organic synthesis, as templates for chiral recognition and as building blocks for polymers and other molecules.^21^, ^22^ These compounds exhibit axial chirality, namely a bond around which rotation is hindered due to large substituents in the closest positions, which are held in a spatial arrangement that is not superimposable to its mirror image.^23^ The photoracemization mechanism of BINOL and its methyl ether was reported by Solntsev et al. in 2009.^24^ They proposed that BINOL derivatives bearing at least a free hydroxyl group reach the excited state upon irradiation with UV light (337 nm), and undergo deprotonation through the so‐called intermolecular excited‐state proton transfer (ESPT) in the presence of a suitable base. The resulting binaphtholate is the key achiral planar intermediate, leading to racemization after reprotonation to the hydroxyl moiety occurs. Inspired by these results we tested the combination of photoinduced racemization and Viedma ripening, and we here demonstrate its successful application to promote the deracemization of a BINOL derivative. This research may pave the way for new Viedma ripening applications to organic conglomerate‐forming compounds that can undergo photochemical racemization.

## Results and Discussion

### Conglomerate screening

**Figure 1:**
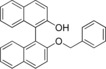
*rac*‐2′‐(Benzyloxy)‐[1,1′‐binaphthalen]‐2‐ol (**1**).

**Figure 2:**
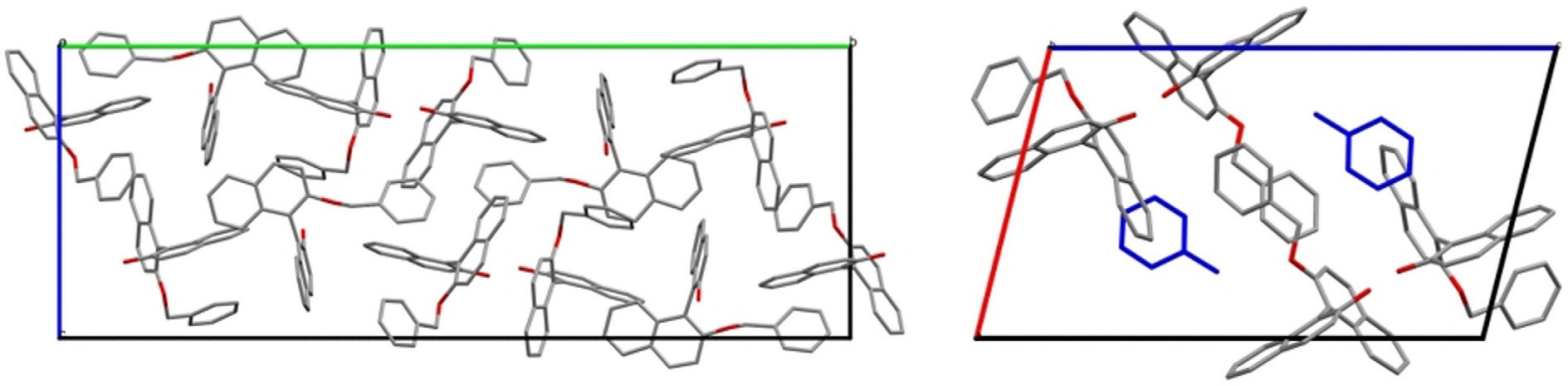
Left: The major conformation of the racemic ansolvate compound form. Right: Crystal packing of the solvate of **1** after crystallization in toluene (blue). Hydrogen atoms are omitted for clarity. The two crystal structures are viewed along the *a*‐axis and *b*‐axis, respectively.

As previously mentioned, one prerequisite for Viedma ripening is that the target compound must crystallize as a conglomerate. Following the choice to use BINOL derivatives, based on previous findings by Solntsev et al,^24^ we focused on finding a conglomerate‐forming target compound. Compound **1**, 2′‐(benzyloxy)‐[1,1′‐binaphthalen]‐2‐ol (Figure 1) was previously reported to manifest conglomerate behavior after crystallization in diethyl ether/hexane.^25^ However, in an attempt to reproduce this experiment, no conglomerate formation was observed by using diethyl ether/hexane as a solvent/antisolvent composition. In fact, the compound crystallized as a new racemic compound form, Figure 2 a.

Therefore, we searched for a crystal form of **1** that does show conglomerate behavior. Recently, our group has reported a systematic approach for predicting suitable coformers to obtain stable cocrystals, some of which potentially being conglomerates.^26^ However, given the complexity that such coformers might add to the system, a convenient and simpler route to explore conglomerate formation was to evaluate a library of different solvents that may give rise to possible conglomerate solvates. Thus far, only one successful Viedma ripening deracemization involving a solvate had previously been reported, although that was a rare case of a conglomerate co‐crystal salt solvate.^27^ Therefore, this work would represent a new example in which Viedma ripening was applied to a conglomerate solvate. To achieve this, a crystallization screening was performed by using approximately thirty solvents. As a result, more than 70 % of the crystallization experiments led to the stable, ansolvate racemic compound form, Figure 2 a. For a phase diagram study of compound **1**, see the report by Hoquante et al.^28^ However, we found that compound **1** forms a conglomerate solvate when crystallization occurred in toluene or chlorobenzene, displaying in both cases a similar crystal structure. As shown in Figure 2 b, two molecules of **1** coordinate one molecule of toluene in the asymmetric unit. For all further racemization and deracemization experiments performed in this work toluene was selected as the solvent of choice.

However, if a slurry of **1** was left longer than 24 hours in toluene, complete conversion to the most stable racemic compound form occurred, which indicates that the conglomerate is in fact metastable. A similar situation was previously reported by Spix et al., who deracemized a metastable conglomerate of glutamic acid using Viedma ripening.^29^

### Racemization study

**Figure 3:**
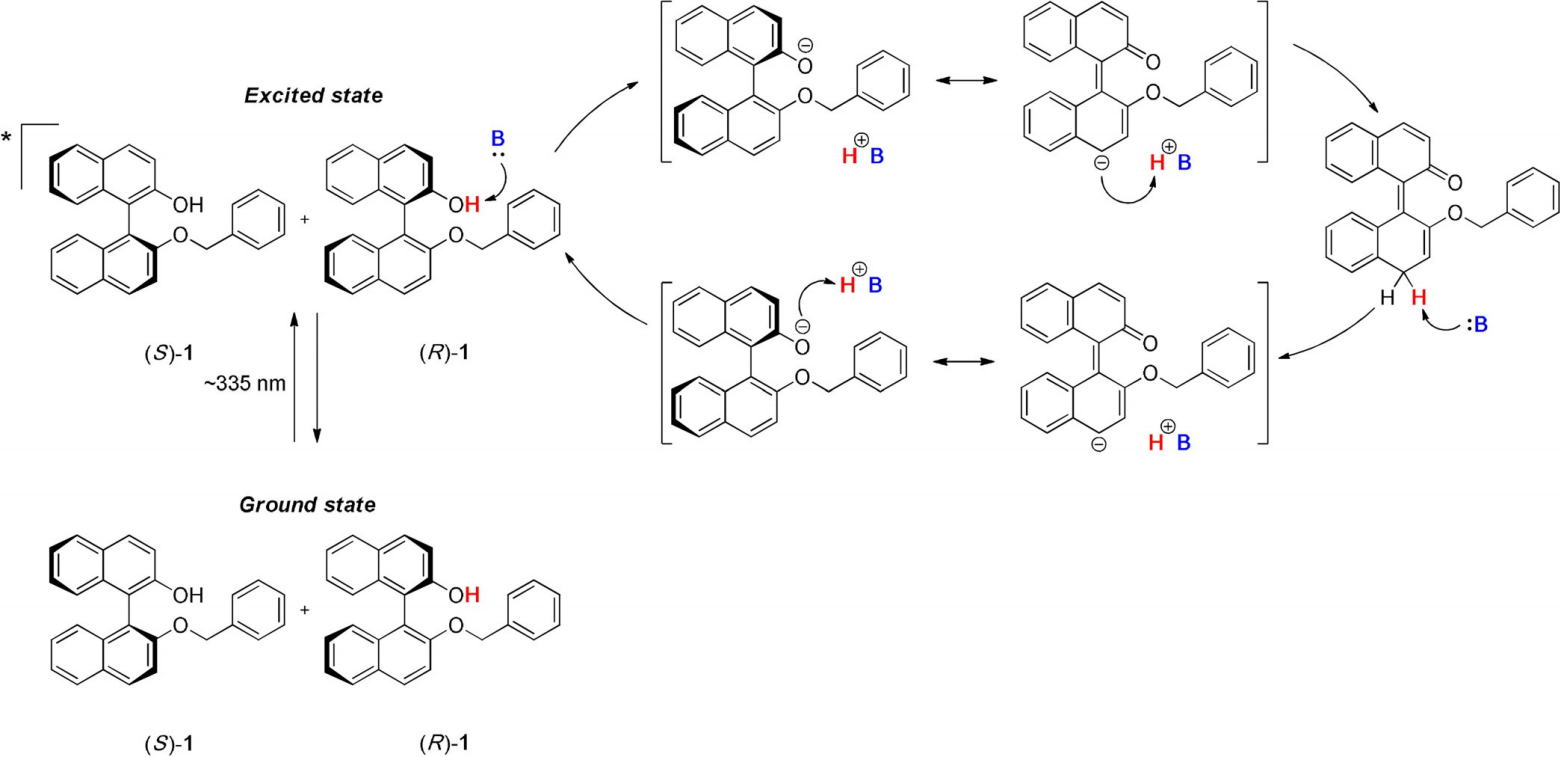
Racemization mechanism proposed for compound **1**. B indicates an organic base.

Xenon, mercury, deuterium and LED lamps were tested as potential light sources for the racemization. Among them, the xenon UV lamp proved to be the best solution, having a broad emission band covering both UV and visible regions. The UV/Vis absorption spectrum for **1** shows mainly three bands: a strong band at 230 nm, and two weaker bands at 285 and 335 nm, as also previously reported by Solntsev et al.^24^ The solvents in which **1** crystallizes as a conglomerate, toluene or chlorobenzene, also absorb in the UV region up to 285 nm (see UV/Vis absorption spectrum in the Supporting Information, Figure S6). Therefore, parallel experiments were performed in both quartz and glass vials, as the latter show a cut‐off of radiation around 300 nm, and the respective effects and differences in the outcome of the reactions were analyzed. Regardless the type of vial used, racemization was observed in all experiments. Recalling the mechanism reported for BINOL and BINOL ethers,^24^ we report here a similar pathway for the target compound **1** (Figure 3).

**Figure 4:**
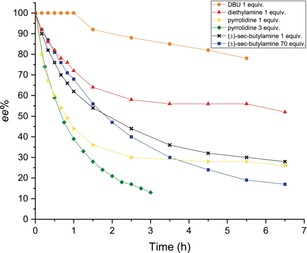
Racemization rates with the use of different bases to promote the ESPT.

Irradiation by UV light promotes compound **1** to its excited state, on which the ESPT can occur. Deprotonation by suitable bases leads to a phenolate charged species, which is in equilibrium with its achiral planar quinoidal form. Subsequently, reprotonation to the hydroxyl moiety occurs, resulting in racemization of the starting enantioenriched material. Some common organic bases, with p*K*_a_ values between 11 and 14, were selected to be screened for their ability to efficiently perform the ESPT. Figure 4 presents an overview of racemization rates observed with different bases. Experiments were performed in toluene in stoichiometric ratio or excess with respect to the substrate by using a xenon lamp as the UV/Vis‐light source. Pyrrolidine and racemic *sec*‐butylamine were finally considered to be the best candidates, most likely because other bases with p*K*a values above 11 are too strong and not suitable for allowing reprotonation to occur.

Two independent experiments using only the base or UV light were performed. No change in the enantiomeric excess was observed, which remained constant at 100 %, demonstrating that the combination of base and UV light is essential for the racemization to occur.

### Deracemization using Viedma ripening

**Figure 5:**
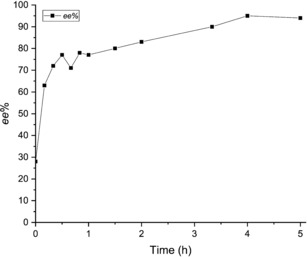
Evolution of the *ee* during a Viedma ripening deracemization experiment. Pyrrolidine was the base used for this experiment.

Full conversion of the metastable conglomerate to the more stable racemic compound occurred in about 24 hours, and thus the Viedma ripening has to take place well within this time frame. Experiments starting from racemic mixtures were not successful, due to the too long deracemization time. Viedma ripening experiments were therefore performed starting from a scalemic mixture (around 20 % *ee*) in order to complete the deracemization more quickly. Figure 5 shows the evolution of the *ee* during one of the Viedma ripening experiments. The deracemization curve displays an initial steep increase of the *ee*, probably because of a rise of the temperature due to the irradiation with UV light. After the temperature stabilizes to a value of about 40 °C, the amplification of the *ee* to reach (nearly) full enantiopurity is decelerated. An *ee* of 96 % was achieved in 4 hours. Analyses of the crystalline phase by SFC (Supercritical Fluid Chromatography), coupled with UV/Vis and mass analyzer detectors, showed that no side products were present. XRPD measurements of the crystalline compound, before and after the experiments, confirmed that during the deracemization the crystals were consistently in their conglomerate form (see Supporting Information). Some of the experiments, however, did not reach a complete enantiopurity probably due to a premature transformation of the conglomerate to the stable racemic compound form. In all cases, the experiments were reproducible and successful both for pyrrolidine and *sec*‐butylamine as the base, and deracemization was achieved in 4–5 hours.

Continued grinding under racemization conditions after deracemization was achieved, however, led to a full conversion to the stable racemic compound, with a consequent drop of the *ee* and loss of enantiopurity. Although ideally stopping the racemization, that is, interrupting the irradiation of the compound by UV light by simply switching off the UV lamp, would be sufficient to preserve enantiopurity of the crystals and allow for their collection, in the present case some small conversion to the racemic compound still occurred. This is due to a crystallization event taking place when the irradiation is stopped and the temperature decreases, allowing for the formation of crystals of the opposite enantiomer and also of the racemic compound. Therefore, an ideal experiment is stopped by 1) harvesting and washing the solid as soon as 100 % *ee* is reached and 2) recrystallizing the final product in a different solvent to allow for the formation of a more stable form for the enantiomerically pure crystals.

In two cases, Viedma ripening experiments were started directly on the crude mixture from the synthesis of **1**, so containing residues such as the starting material (BINOL) and the disubstituted side product. In presence of these “impurities”, the experiments proceeded to completion in a much shorter time, leading in both cases to the final compound with (*R*)‐configuration. Steendam et al. already showed that the presence of small, chiral impurities can steer the outcome of the deracemization and can speed up the process.^30^ As recently reported, a very small amount of chiral impurities can already be sufficient to halve the deracemization time.^31^

## Conclusions

We have reported here the first example of a photoassisted Viedma ripening deracemization using UV light. The compound investigated in this paper proved to be a metastable conglomerate solvate. A xenon lamp was the optimum light source because it has a broad emission spectrum covering all UV absorption regions of the compound. Several bases were evaluated for their ability to promote the subsequent ESPT, necessary for the racemization to occur. Unlike a typical Viedma ripening experiment, an initial enantiomeric excess was required to speed up the process to completion before the change of the metastable conglomerate crystal phase to the stable racemic compound form occurred. This shows the importance of detecting the different crystal forms of a specific compound when dealing with possible metastable phases. Chiral impurities might be very helpful in the presence of metastable conglomerates, when deracemization has to be completed in a short time frame. The application of Viedma ripening may be extended to other conglomerate‐forming chiral organic compounds that show racemization under UV light irradiation.

## Experimental Section

### Synthesis of the starting material

Racemic BINOL, (*R*)‐BINOL, K_2_CO_3_ and benzyl bromide were purchased from Sigma–Aldrich and used as received. Racemic and enantiopure 2′‐(benzyloxy)‐[1,1′‐binaphthalen]‐2‐ol were synthesized following a reported procedure.^32^ Racemic BINOL (500 mg, 1.75 mmol) or (*R*)‐BINOL (500 mg, 1.75 mmol) was dissolved in ca. 15 mL of acetone, before K_2_CO_3_ (2.62 mmol, 1.5 equiv) and benzyl bromide (1.75 mmol, 1 equiv) were added. The solution was heated up to 60 °C for about 4 h until all the starting material was consumed, monitored with TLC. The crude mixture was then concentrated under reduced pressure and extracted with ethyl acetate (3×20 mL) and water. The organic phases were collected, dried with magnesium sulfate and concentrated under reduced pressure. Column chromatography (heptane/ethyl acetate 8:2) was lastly used to collect the final product, recrystallized in toluene to give a white powder (yield: 80 %). ^1^H NMR (400 MHz, chloroform‐*d*) *δ*=7.91 (d, *J=*9.0 Hz, 1 H), 7.87–7.83 (m, 1 H), 7.80 (dt, *J=*8.2, 0.9 Hz, 2 H), 7.39 (d, *J=*9.1 Hz, 1 H), 7.33–7.26 (m, 2 H), 7.24 (dt, *J=*6.8, 1.3 Hz, 1 H), 7.20 (dd, *J=*6.7, 1.4 Hz, 1 H), 7.15 (dddd, *J=*8.2, 3.8, 3.0, 1.4 Hz, 2 H), 7.10 (dt, *J=*4.3, 1.6 Hz, 3 H), 7.05–6.92 (m, 3 H), 5.12–4.93 (m, 2 H), 4.85 ppm (s, 1 H); ^13^C NMR (100 MHz, chloroform‐*d*) *δ*=154.94, 151.29, 136.92, 134.06, 133.82, 130.86, 129.83, 129.68, 129.15, 128.33, 128.13, 128.10, 127.65, 127.29, 126.89, 126.41, 125.04, 124.95, 124.43, 123.24, 117.50, 116.78, 115.94, 115.08, 77.02, 76.70, 71.13 ppm. XRPD diffractograms of the racemic and the enantiopure form were recorded by using a Bruker D8 Advance Diffractometer (see Supporting Information).

### Racemization experiments

Experiments were performed at room temperature in 20 mL glass vials with cutoff of radiation at 300 nm, as well as quartz vials. 25 mg of (*R*)‐**1** were dissolved in 3 mL of toluene, after which 1 equiv of the base was added to promote the “proton transfer” mechanism, and therefore the racemization. The solution was irradiated with a UV xenon lamp (150 W) and samples were taken every 10 minutes during the first hour, and subsequently every 30 minutes. The lamp was positioned at a distance of about 20 cm from the vial used, to prevent a drastic increase in the temperature at which the experiments took place. The temperature measured during the experiments ranged from an initial 22 °C to a steady value of 40 °C. The enantiomeric excess was measured by using a Waters SFC (Supercritical Fluid Chromatography), equipped with both UV and mass spectrometer detectors, on a LUX amylose‐1 column with a flow rate of 0.5 mL min^−1^. Retention times were 2.2 min and 3.2 min for the (*S*)‐ and (*R*)‐enantiomers, respectively. Chromatograms were recorded at a wavelength of 230 nm, corresponding to the maximum absorption peak of the BINOL derivative.

### Deracemization experiments

The experiments were performed at room temperature in 20 mL glass vials with cut‐off of radiation at 300 nm, as well as quartz vials. An initial enantiomeric excess (ca. 20 %) was necessary to ensure that the deracemization process would reach completion before the conversion to the more stable racemic compound crystals took place. A slurry was made from 1.2 g of *rac*‐**1**, 0.3 g of (*R*)‐**1** and 1.5 mL of toluene. Following a homogenization time of about 15 minutes, 50 μL of the chosen base and 1 g Ø 2 mm glass beads were added. The suspension was stirred at 700 rpm and then subjected to UV‐light exposure by using a UV xenon lamp (150 W). The lamp was positioned at a distance of about 20 cm from the vial used and the temperature measured during the experiments ranged from 22 °C to a steady value of 40 °C. Placing the lamp closer to the vial resulted in complete dissolution of all solid material, due to the heat produced by the lamp (nearly 50 °C). Samples were collected every 10 minutes for the first hour and then every 30 minutes, filtered off directly from the reaction slurry and analyzed after being carefully washed with one or two drops of toluene. At the end of the experiments, roughly 0.45 g of enantiopure material was collected, which represents a net gain of half of the amount of enantiopure compound initially added. The enantiomeric excess was measured by using a Waters SFC (Supercritical Fluid Chromatography), equipped with both UV and mass spectrometer detectors, on a LUX amylose‐1 column with a flowrate of 0.5 mL min^−1^. Retention times were 2.2 min and 3.2 min for the (*S*)‐ and (*R*)‐enantiomers, respectively. Chromatograms were recorded at a wavelength of 230 nm, corresponding to the maximum absorption peak of the BINOL derivative. No peaks of possible side products forming during the exposure time were detected after SFC analyses. Characterization of the final enantiopure product with XRPD showed an identical diffractogram as for the starting material, indicating that the experiments were stopped in time before the crystals would undergo any conversion to the racemic compound form. XRPD diagrams of all solid samples collected during the deracemization experiments were also analyzed, to prove that the toluene solvate compound was not converted.

CCDC https://www.ccdc.cam.ac.uk/services/strctures?id=doi:10.1002/chem.201904382 contain the supplementary crystallographic data for this paper. These data are provided free of charge by http://www.ccdc.cam.ac.uk/.

## Conflict of interest

The authors declare no conflict of interest.

## Supporting information

As a service to our authors and readers, this journal provides supporting information supplied by the authors. Such materials are peer reviewed and may be re‐organized for online delivery, but are not copy‐edited or typeset. Technical support issues arising from supporting information (other than missing files) should be addressed to the authors.

Supplementary
