# Supplementary material for: Photoracemization‐Based Viedma Ripening of a BINOL Derivative
Source: Chemistry. 2019 Dec 12;26(4):839–44. doi: 10.1002/chem.201904382 (PMC7004087; doi:10.1002/chem.201904382)
Supplement: Supplementary file 1 — Supplementary [file CHEM-26-839-s001.pdf]

CHEMISTRY
A European Journal

Supporting Information

Photoracemization-Based Viedma Ripening of a BINOL Derivative**

Giuseppe Belletti+,[a] Carola Tortora+,[b] Indradevi D. Mellema,[a] Paul Tinnemans,[a] Hugo Meekes,[a] Floris P. J. T. Rutjes,[a] Svetlana B. Tsogoeva,*[b] and Elias Vlieg*[a]

chem_201904382_sm_miscellaneous_information.pdf

DSC curves

The different crystal forms detected were subjected to DSC studies. Below is the overview of the melting points of all of them.

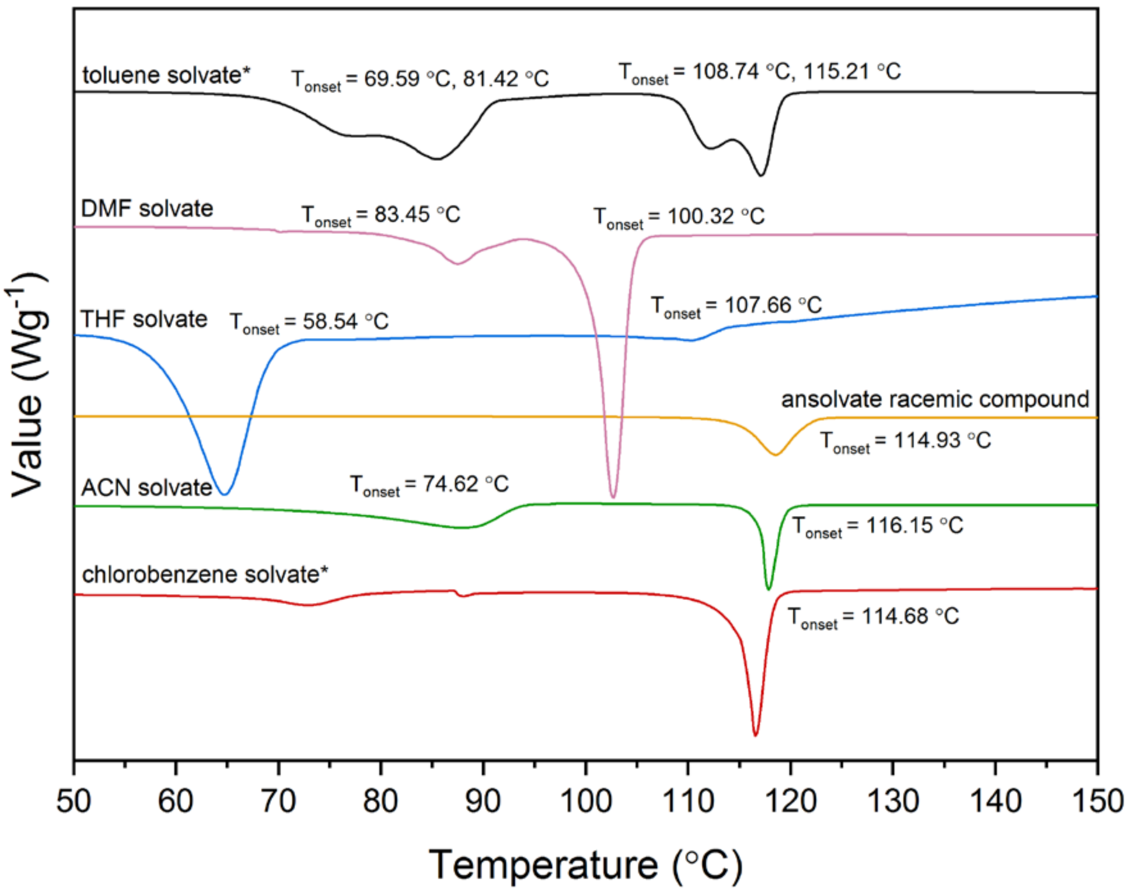

Figure 1. DSC curves of the different crystal forms of 1. * indicates the conglomerates. Scan rate: 2°C/min.

Figure 2 displays the DSC curves before and after Viedma ripening. Conversion to the racemic compound occurred when the experiments were left longer than 24h in solution. DSC analyses were taken at the beginning of the deracemization process, at the moment enantiopurity was achieved (this particular curve was collected at 90% *ee*) and at the end of the experiment, after the crystal transformation occurred (*ee* dropped to 0%).

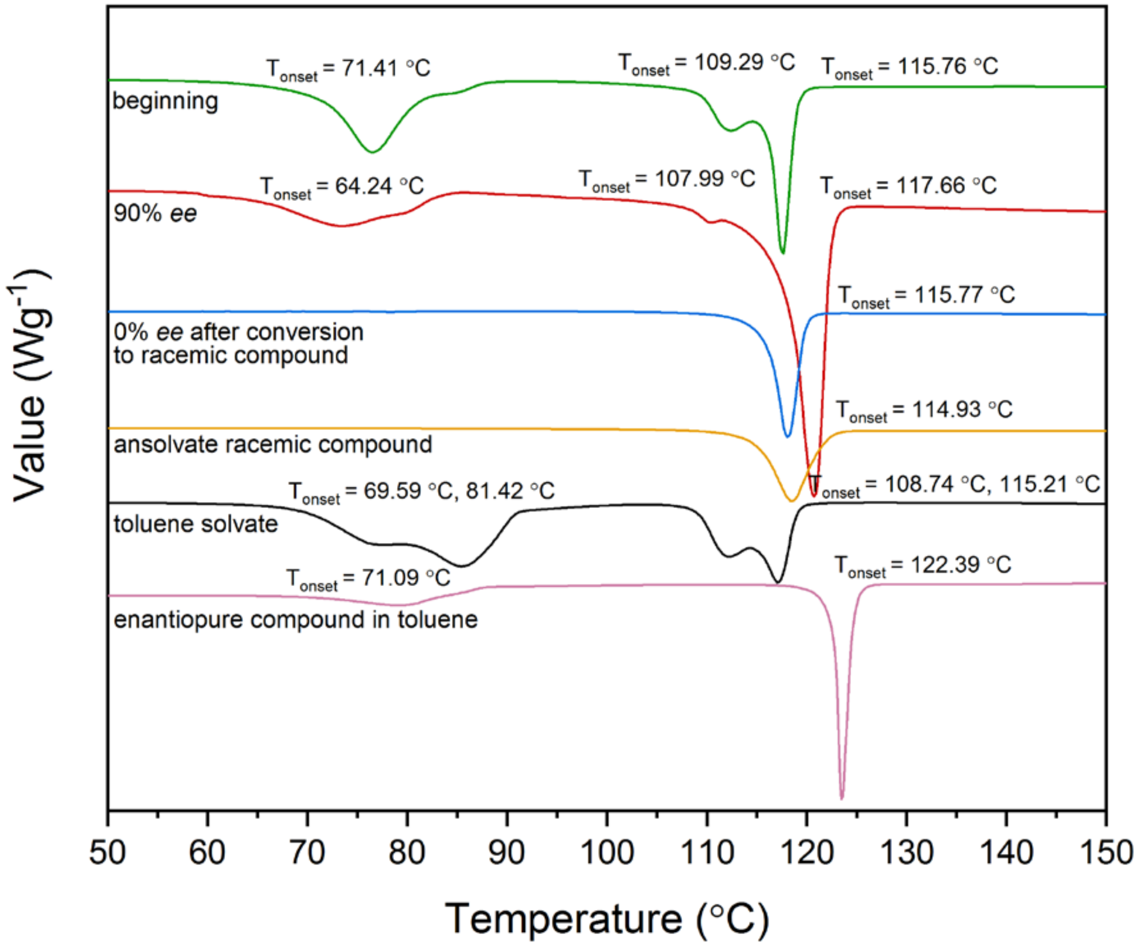

Figure 2. DSC measurements of **1** at the beginning of the process, at completion of the deracemization (90% *ee*) and at the end of the process (*ee* dropped to 0% due to conversion to racemic compound). At the bottom: references of the conglomerate toluene solvate, in its racemic and enantiopure form, and of the ansolvate racemic compound. Scan rate: 2°C/min.

XRPD diffractograms

To prove that the compound of interest was a conglomerate when crystallized from toluene, XRPD diffractograms of the racemic mixture and the enantiopure 2'-(benzyloxy)-[1,1'-binaphthalen]-2-ol were recorded, and they are shown in Figure 3. XRPD diffractograms of the racemic and the enantiopure form were obtained using a Bruker D8 Advance Diffractometer.

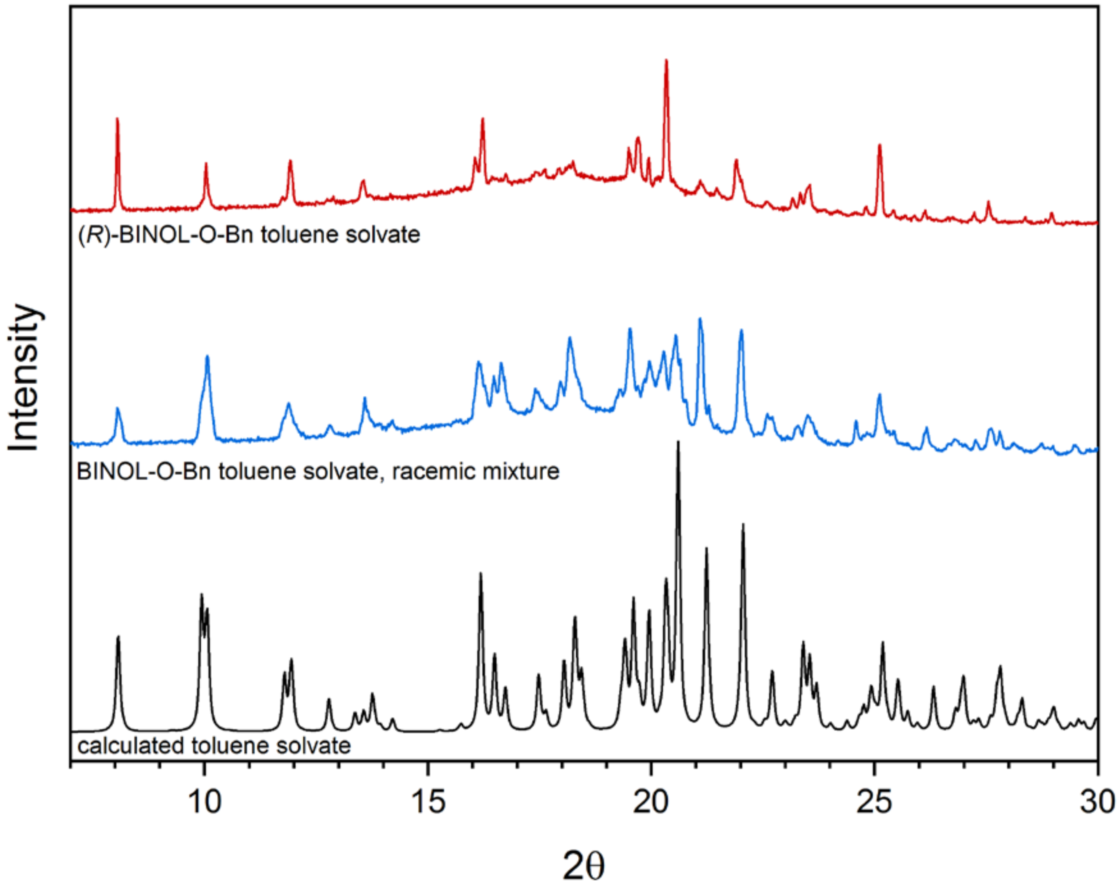

Figure 3. XRPD diffractograms of the enantiopure (red) and a racemic mixture of **1** (blue) 2'-(benzyloxy)-[1,1'-binaphthalen]-2-ol. In black, the calculated diffractograms from the single-crystal analysis of the conglomerate **1**.

XPRD diffractograms were also collected during Viedma ripening, to show the full conversion of **1** to the stable racemic compound form, after enantiopurity was reached. In particular, Figure 4 shows XRPD analyses taken at the beginning of the deracemization process, at the moment enantiopurity was achieved (this particular diffractogram was collected at 90% *ee*) and at the end of the experiment, after the crystal transformation occurred (*ee* dropped to 0%).

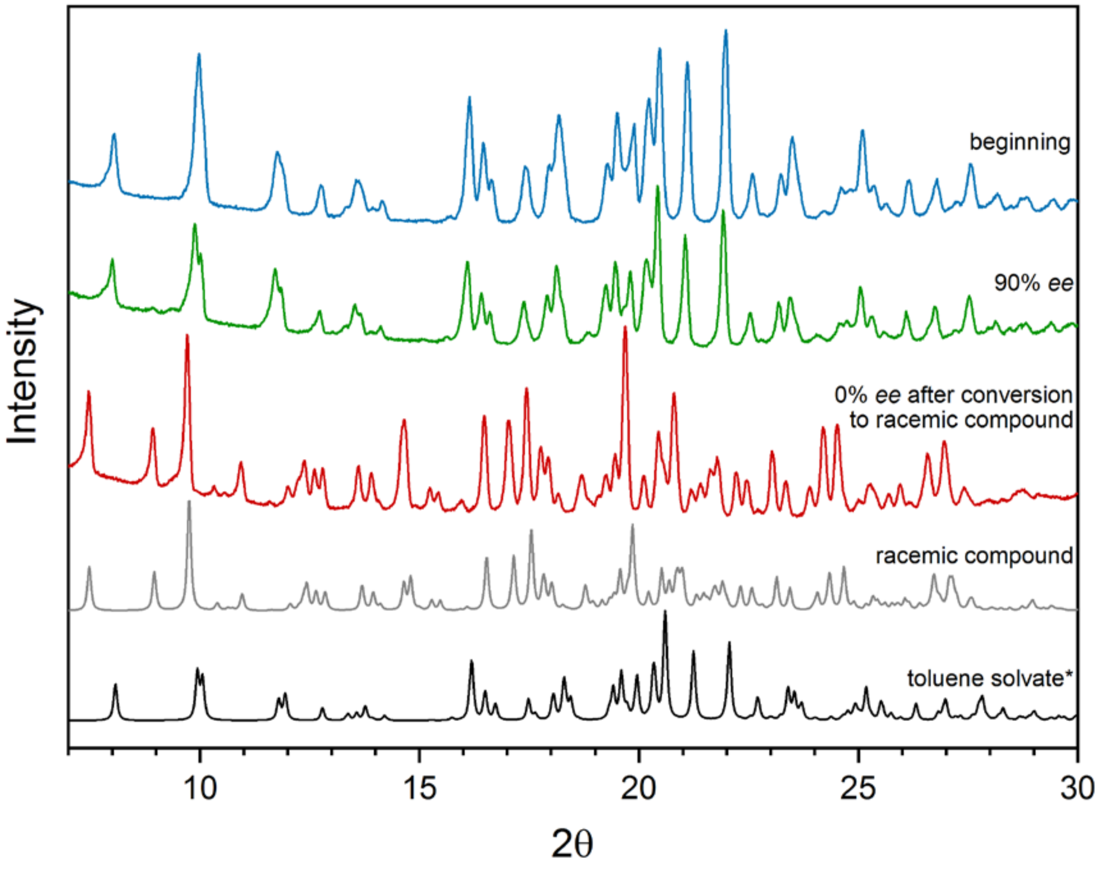

Figure 4. XRPD diffractograms of **1** at the beginning of the process, at completion of the deracemization (90% *ee*) and at the end of the process (*ee* dropped to 0% due to conversion to racemic compound). At the bottom: references of the ansolvate racemic compound form and the conglomerate solvate. * indicates the conglomerate.

To avoid the crystal transformation and the loss of enantiopurity, crystals should be filtered off and dried as soon as they reach 100% *ee*. Recrystallization in another solvent would help recovering the enantiopure compound in a more stable form.

SFC measurements

One of the proves that no degradation of the compound occurred after UV-light exposure is shown below. The SFC analysis, with the aid of a mass spectrometry detector, reveals that the mass of the compound does not change during the Viedma ripening deracemization.

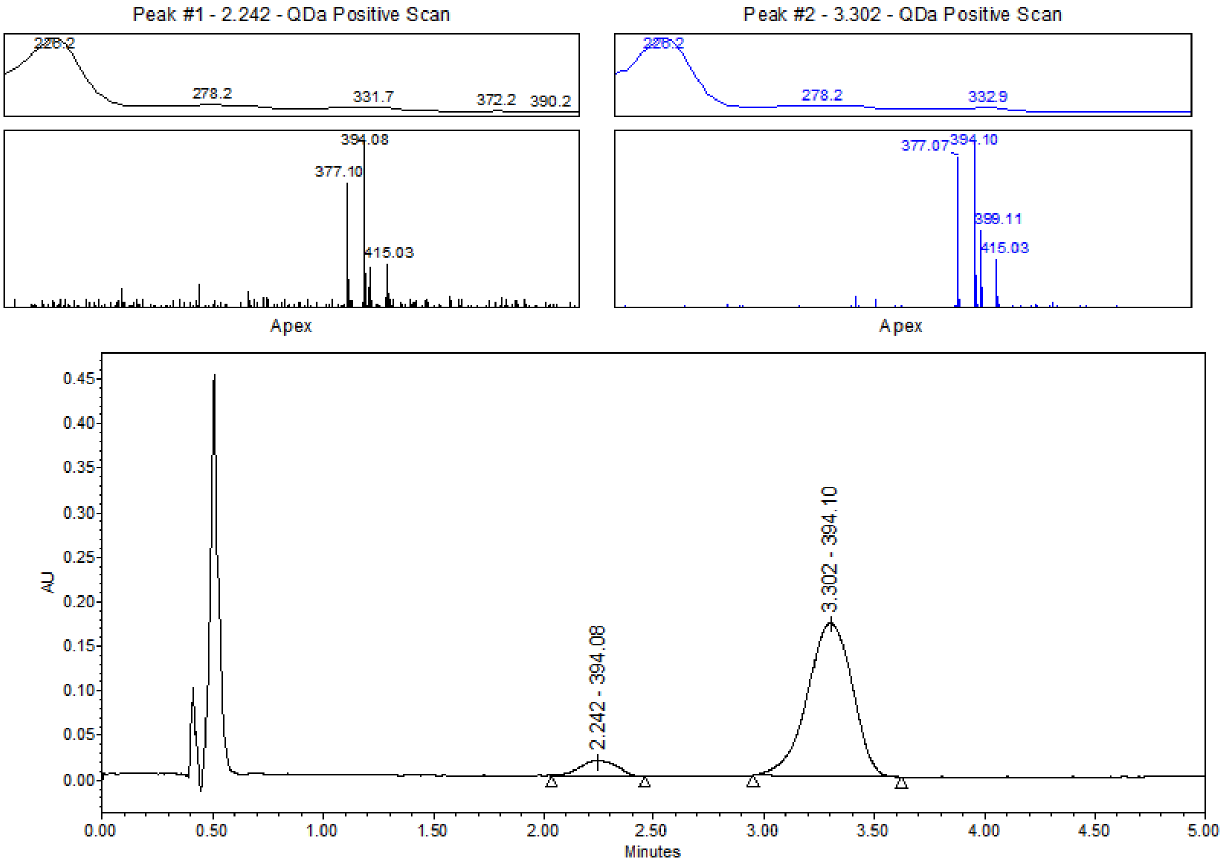

Figure 5. Example of a SFC analysis during Viedma ripening (displayed *ee* = 86%). Top: mass spectra of the peaks of the two enantiomers (BINOL-O-Bn: 376 m/z; ion adducts from the ESI mass spectrometry technique: $H^{+:}$ 377 m/z, NH_4^+: 394 m/z, Na^+: 399 m/z, K^+: 415 m/z). Bottom: SFC chromatogram showing the (*S*) and the (*R*) enantiomer respectively. Retention times: 2.2 min for (*S*) and 3.2 min for (*R*).

Absorption spectrum

Figure 6 shows the absorption spectrum of **1** together with toluene and chlorobenzene. The light grey region represents the part of the spectrum which becomes inactive when glass vials are used (cut-off wavelength of glass is 300 nm).

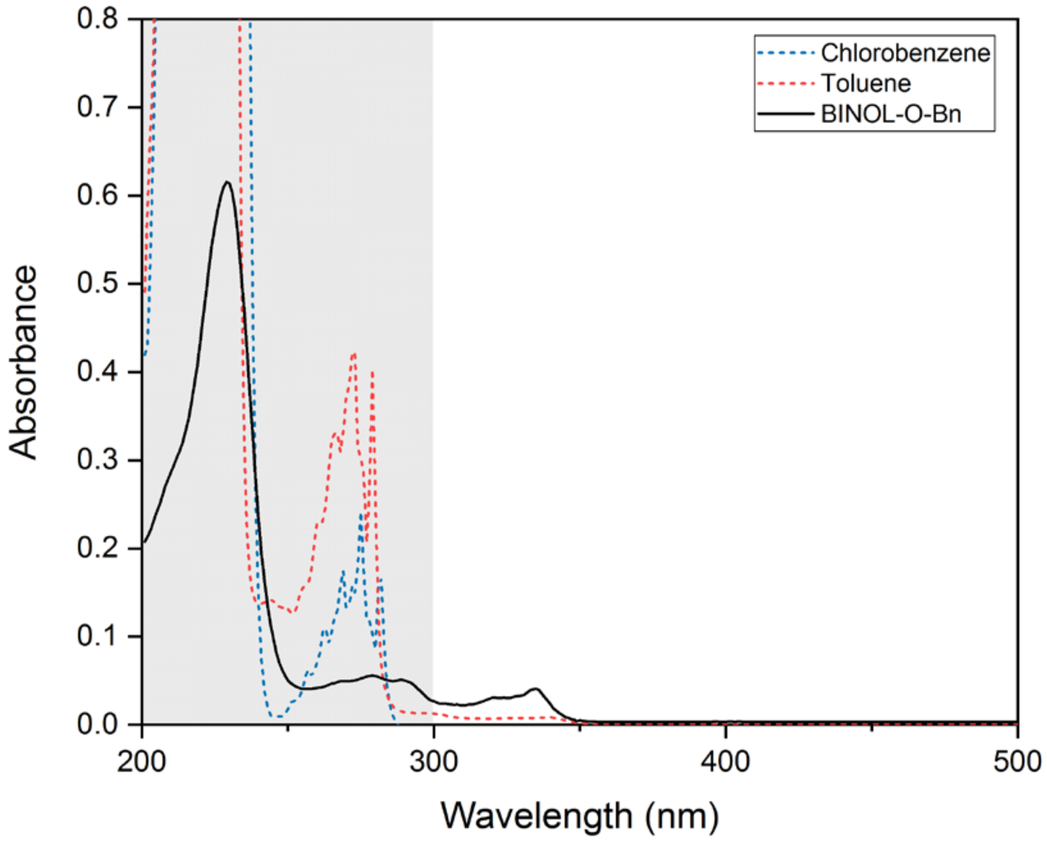

Figure 6. Absorption spectrum of **1** (black line), toluene (red dotted line) and chlorobenzene (blue dotted line). The data are plotted on the same relative scale. As shown, both the solvents do not absorb light for values >300 nm. The grey region represents the part of the absorption spectrum which is inactive with the use of glass vials. For glass the cut-off wavelength is 300 nm.

Single-crystal analyses

Single-crystal analyses were performed using a Bruker D8 Quest Apex3. The .cif files of all the crystal forms are uploaded together with this manuscript. All crystal forms have been submitted on the CCDC (Deposition Number 1940976-1940981).

Toluene solvate, conglomerate

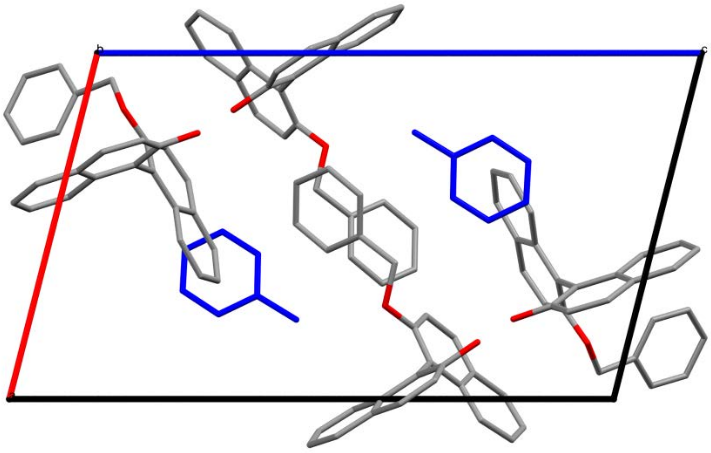

Figure 7. Toluene conglomerate solvate (viewed along the b-axis). Hydrogen atoms are omitted for clarity. Solvent is drawn in blue. Crystal system: monoclinic. Space group: $P2_1$.

Ansolvate form, racemic compound (major conformation)

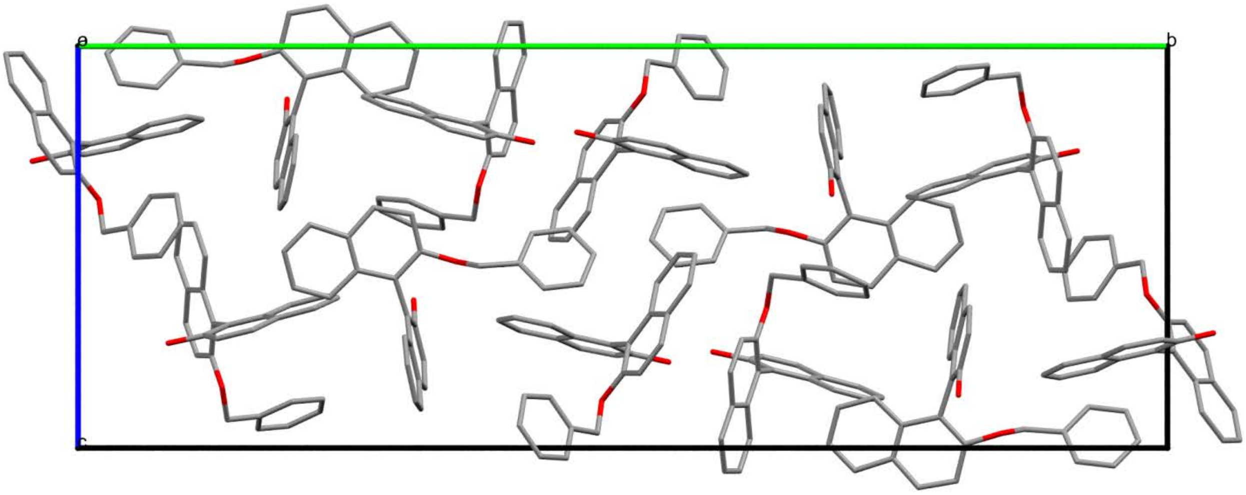

Figure 8. The major conformation of the racemic ansolvate compound form (viewed along the a-axis). Hydrogen atoms are omitted for clarity. Crystal system: monoclinic. Space group: $P2_1/n$.

Acetonitrile solvate, racemic compound

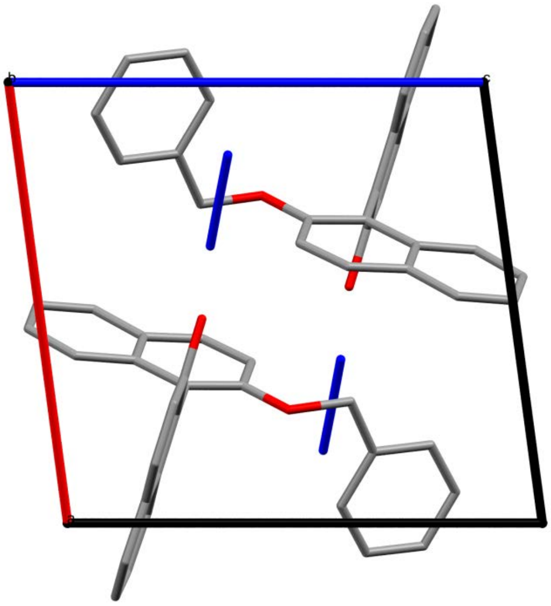

Figure 9. Acetonitrile racemic compound solvate (viewed along the b-axis). Hydrogen atoms are omitted for clarity. Solvent is drawn in blue. Crystal system: triclinic. Space group: *P*-1.

THF solvate, racemic compound (major conformation)

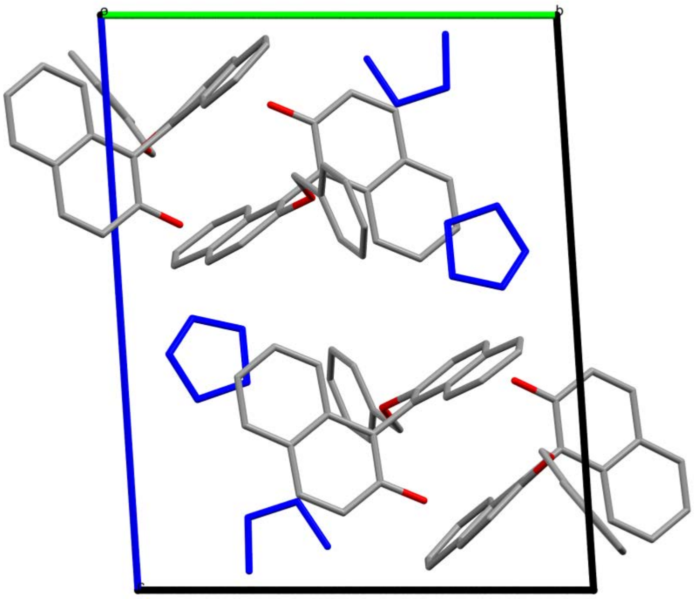

Figure 10. The major conformation of the THF racemic compound solvate (viewed along the a-axis). Hydrogen atoms are omitted for clarity. Solvent is drawn in blue. Crystal system: triclinic. Space group: *P*-1.

DMF solvate, racemic compound

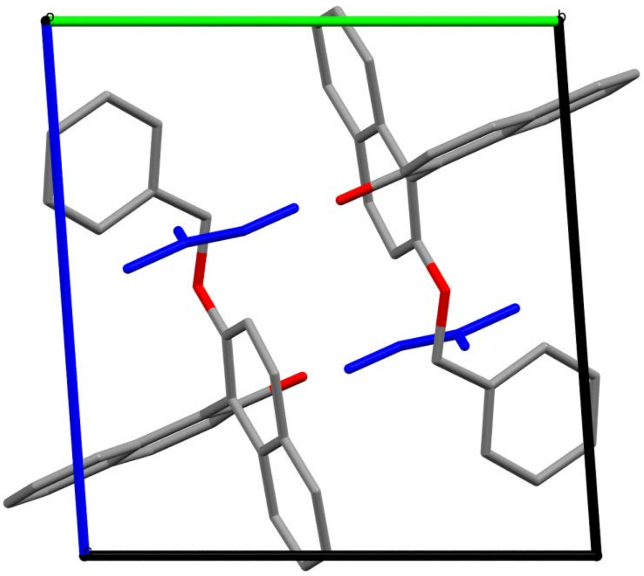

Figure 11. DMF racemic compound solvate (viewed along the a-axis). Hydrogen atoms are omitted for clarity. Solvent is drawn in blue. Crystal system: triclinic. Space group: *P*-1.

Chlorobenzene solvate, conglomerate

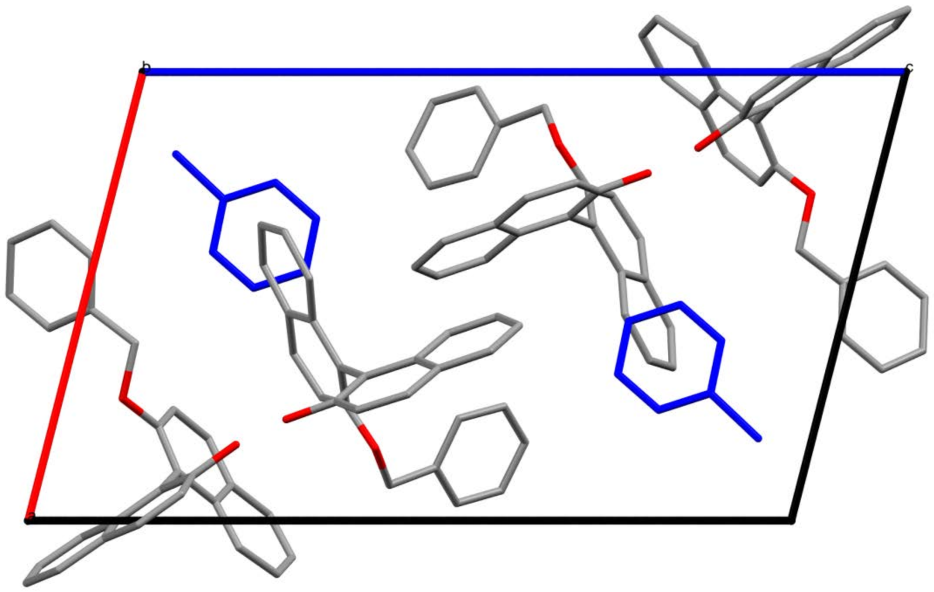

Figure 12. Chlorobenzene conglomerate solvate (viewed along the b-axis). Hydrogen atoms are omitted for clarity. Solvent is drawn in blue. Crystal system: monoclinic. Space group: $P2_1$.
